# Supplementary material for: A retrospective evaluation of preemptive liver transplantation for bile duct dysplasia in primary sclerosing cholangitis: Balancing risks and benefits
Source: JHEP Rep. 2025 Sep 20;7(12):101598. doi: 10.1016/j.jhepr.2025.101598 (PMC12657721; doi:10.1016/j.jhepr.2025.101598)
Supplement: Multimedia component 1 [file mmc1.pdf]

A retrospective evaluation of preemptive liver transplantation for bile duct dysplasia in primary sclerosing cholangitis: Balancing risks and benefits

Sigurd Breder, Christina Villard, Emma Eide, Benny Wang, Lise Katrine Engesæter, Henrik Mikael Reims, Johannes Roksund Hov, Espen Melum, Lars Aabakken, Pål Dag Line, Jon Lømo, Krzyztof Grzyb, Kristine Wiencke, Annika Bergquist, Trine Folseraas

Table of contents

Fig. S1 2

Table S1 3

Fig. S1. The survival distribution in cholangiocarcinoma up to 10-years following liver transplantation divided according to stage

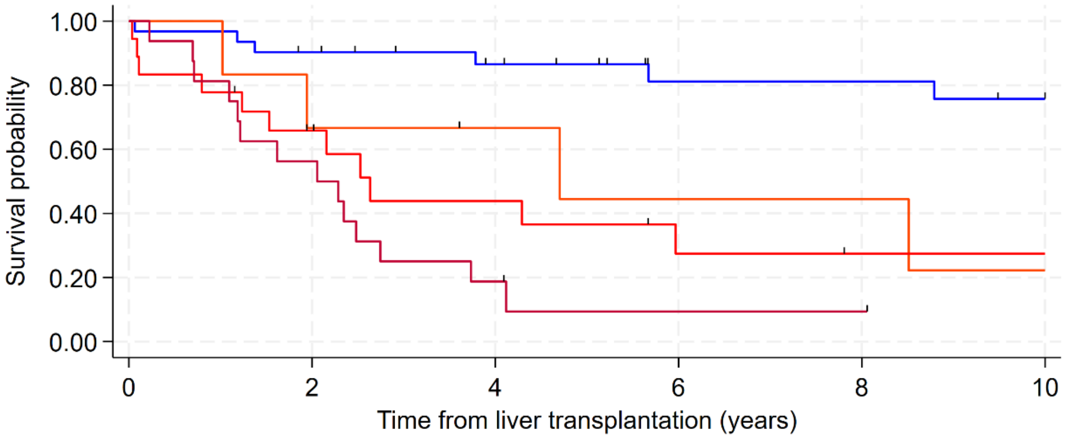

Number at risk

	0	2	4	6	8	10
HGD/Stage 0 CIS	31	27	22	15	15	13
CCA stage 1	6	4	3	2	2	1
CCA stage 2	18	9	6	3	2	2
CCA stage 3-4	16	9	3	1	1	0

HGD/Stage 0 CIS — Stage 1 — Stage 2 — Stage 3-4

CCA: Cholangiocarcinoma. HGD: High-grade dysplasia. CIS: Carcinioma in situ. Stage according to The American Joint Committee on Cancer (AJCC) TNM-system 8th edition

Table S1. Causes of death, stratified by primary indication for liver transplantation in primary sclerosing cholangitis

	All patients (n = 512) (%)	Primary indication for listing to liver transplantation			
		Low-grade biliary dysplasia (n = 29) (%)	High-grade biliary dysplasia (n = 59) (%)	Suspicion of cholangiocarcinoma (n = 40) (%)	All other indications (n = 384) (%)
**Dead (by December 31st 2022)**	106 (20.7)	3 (10.3)	12 (20.3)	21 (52.5)	70 (18.2)
**Age at death, years (range)**	54.8 (25.4-82.8)	48.8 (40.1-65.4)	54.5 (44.1-71.5)	54.8 (42.1-76.6)	54.8 (25.4-82.8)
**Time from LT to death, years (range)**	3.8 (0-22.5)	7.4 (0.04-18.6)	2.4 (0.07-19.3)	2.3 (0.1-13.9)	5.0 (0-22.5)
**Cause of death**					
PSC-related malignancy	34 (32.1)	0	5 (41.6)	14 (66.6)	15 (21.4)
Transplant complications	24 (22.6)	1 (33.3)	2 (16.7)	2 (9.5)	19 (27.1)
Other	12 (11.3)	1 (33.3)	2 (16.7)	3 (14.3)	6 (8.6)
Other malignancy	9 (8.5)	0	1 (8.3)	1 (4.8)	7 (10.0)
Recurrent PSC	3 (2.8)	0	0	0	3 (4.3)
Unknown	24 (22.7)	1 (33.3)	2 (16.7)	1 (4.8)	20 (28.6)

Values are presented as n (%), median (range). All percentages are presented as valid percent. LT: Liver transplantation.
