# Supplementary material for: A retrospective evaluation of preemptive liver transplantation for bile duct dysplasia in primary sclerosing cholangitis: Balancing risks and benefits
Source: JHEP Rep. 2025 Sep 20;7(12):101598. doi: 10.1016/j.jhepr.2025.101598 (PMC12657721; doi:10.1016/j.jhepr.2025.101598)
Supplement: Multimedia component 2 [file mmc2.docx]

**Journal of Hepatology**

**CTAT methods**

Tables for a “Complete, Transparent, Accurate and Timely account” (CTAT) are now mandatory for all revised submissions. The aim is to enhance the reproducibility of methods.

- Only include the parts relevant to your study
- Refer to the CTAT in the main text as ‘Supplementary CTAT Table’
- Do not add subheadings
- Add as many rows as needed to include all information
- Only include one item per row

**If the CTAT form is not relevant to your study, please outline the reasons why:**

| The CTAT form is not relevant to our study.  The study is a retrospective cohort study evaluating results of bile duct brush cytology and intraductal biopsies and compared with histology of explanted liver following liver transplantation in primary sclerosing cholangitis.  We have not used any antibodies, cell lines, organisms, sequence based reagents, biological samples, deposited data or other in this study. We performed statistical analyses using STATA/SE version 17.0 (StataCorp LLC, TX, USA), as noted in the manuscript. Biorender was used for creating the graphical abstract. |
| --- |

- 1. **Antibodies**

| **Name** | **Citation** | **Supplier** | **Cat no.** | **Clone no.** |
| --- | --- | --- | --- | --- |
|  |  |  |  |  |

- 1. **Cell lines**

| **Name** | **Citation** | **Supplier** | **Cat no.** | **Passage no.** | **Authentication test method** |
| --- | --- | --- | --- | --- | --- |
|  |  |  |  |  |  |

- 1. **Organisms**

| **Name** | **Citation** | **Supplier** | **Strain** | **Sex** | **Age** | **Overall n number** |
| --- | --- | --- | --- | --- | --- | --- |
|  |  |  |  |  |  |  |

- 1. **Sequence based reagents**

| **Name** | **Sequence** | **Supplier** |
| --- | --- | --- |
|  |  |  |

- 1. **Biological samples**

| **Description** | **Source** | **Identifier** |
| --- | --- | --- |
|  |  |  |

- 1. **Deposited data**

| **Name of repository** | **Identifier** | **Link** |
| --- | --- | --- |
|  |  |  |

- 1. **Software**

| **Software name** | **Manufacturer** | **Version** |
| --- | --- | --- |
| Statistical analyses were performed using STATA/SE  Biorender | StataCorp LLC, TX, USA  Biorender | version 17.0 |

- 1. **Other (e.g. drugs, proteins, vectors etc.)**

|  |  |  |
| --- | --- | --- |
|  |  |  |

- 1. **Please provide the details of the corresponding methods author for the manuscript:**

| **Sigurd Breder** |
| --- |

**2.0 Please confirm for randomised controlled trials all versions of the clinical protocol are included in the submission. These will be published online as supplementary information.**

|  |
| --- |
