# Supplementary material for: A retrospective evaluation of preemptive liver transplantation for bile duct dysplasia in primary sclerosing cholangitis: Balancing risks and benefits
Source: JHEP Rep. 2025 Sep 20;7(12):101598. doi: 10.1016/j.jhepr.2025.101598 (PMC12657721; doi:10.1016/j.jhepr.2025.101598)
Supplement: Multimedia component 4 [file mmc4.pdf]

Research article

JHEP Reports

A retrospective evaluation of preemptive liver transplantation for bile duct dysplasia in primary sclerosing cholangitis: Balancing risks and benefits

Authors

Sigurd Breder, Christina Villard, Emma Eide, …, Kristine Wiencke, Annika Bergquist, Trine Folseraas

Correspondence

trine.folseraas@medisin.uio.no (T. Folseraas).

Graphical abstract

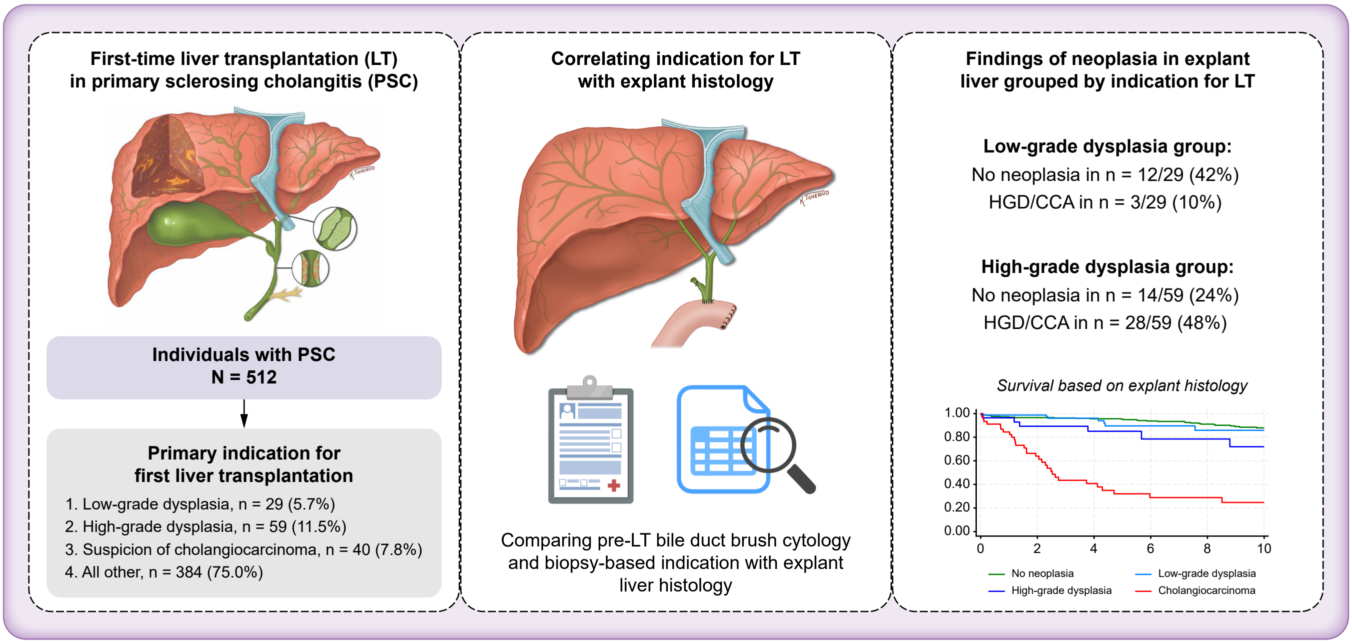

Highlights:

- Liver transplantation for PSC with bile duct dysplasia may prevent cholangiocarcinoma.
- No neoplasia in 42% of explants in those transplanted due to low-grade dysplasia.
- Survival after LT is excellent in individuals with both low- and high-grade dysplasia.
- Low-grade dysplasia calls for an individualized assessment before considering LT.
- Liver transplantation is justified in individuals with high-grade dysplasia.

Impact and implications:

Individuals with primary sclerosing cholangitis (PSC) are at increased risk of developing bile duct cancer. In precancerous stages (bile duct dysplasia), liver transplantation (LT) may prevent progression to advanced, incurable cancer. In our evaluation of 512 patients with PSC who underwent LT, we found that the low diagnostic accuracy and unpredictable detection of mild dysplasia support a cautious, individualized approach to LT in precancerous stages of PSC, while more advanced dysplasia remains a valid indication for LT.

https://doi.org/10.1016/j.jhepr.2025.101598
© 2025 The Authors. Published by Elsevier B.V. on behalf of European Association for the Study of the Liver (EASL). This is an open access article under the CC BY license (http://creativecommons.org/licenses/by/4.0/). JHEP Reports, 2025, 7, 1–10

A retrospective evaluation of preemptive liver transplantation for bile duct dysplasia in primary sclerosing cholangitis: Balancing risks and benefits

Sigurd Breder[1,2,†], **Christina Villard**[3,4,†], Emma Eide[1], Benny Wang[3], Lise Katrine Engesæter[1,2,5], Henrik Mikael Reims[6], Johannes Roksund Hov[1,2,5,7], Espen Melum[1,2,5,7,8], Lars Aabakken[1,2,5], Pål Dag Line[1,9], Jon Lømo[6], Krzyztof Grzyb[6], Kristine Wiencke[5], Annika Bergquist[4,10], Trine Folseraas[1,2,5,*]

JHEP Reports **2025**. vol. 7 | 1–10

Check for updates

Background & Aims: Liver transplantation (LT) to prevent cholangiocarcinoma (CCA) in individuals with primary sclerosing cholangitis (PSC) and bile duct dysplasia was introduced in Norway and Sweden in the early 2000s. We aimed to evaluate this practice to potentially improve future selection of candidates for LT.

Methods: We conducted a retrospective study of 512 adults with PSC who underwent first-time LT between 2000–2021 at Oslo and Karolinska University Hospitals. Pre-LT findings in bile duct brush cytology and/or biopsy of low-grade dysplasia (LGD) and high-grade dysplasia (HGD) were compared with histological findings in the explanted livers and survival rates were assessed.

Results: Bile duct dysplasia, low-grade (LGD) or high-grade (HGD), was the primary LT indication in 17% (88/512). Among individuals transplanted for LGD, only 10% (3/29) had HGD or CCA in the explant, compared to 48% (28/59) in the HGD group. No neoplasia was found in 42% (12/29) of LGD and 24% (14/59) of HGD cases, meaning nearly one-third of patients transplanted for suspected bile duct dysplasia had no histological evidence of neoplasia in the explant. Five-year post-transplant survival according to the explant histology was 95% for no neoplasia, 89% for LGD, 85% for HGD, decreasing to 33% in those with CCA in the explant.

Conclusions: While favorable survival in confirmed dysplasia supports the role of preemptive LT, the absence of neoplasia in a substantial proportion of explants, particularly in suspected LGD, calls for a cautious, individualized approach. LT appears more clearly justified in accurately diagnosed HGD, given its strong association with early malignancy and the poor prognosis of advanced CCA.

© 2025 The Authors. Published by Elsevier B.V. on behalf of European Association for the Study of the Liver (EASL). This is an open access article under the CC BY license (http://creativecommons.org/licenses/by/4.0/).

Introduction

Primary sclerosing cholangitis (PSC) is a chronic progressive bile duct disease characterized by multifocal inflammatory and fibrotic biliary strictures and gradual development into end-stage liver disease.[1] Individuals with PSC are at a high risk of developing cholangiocarcinoma (CCA) with a reported annual incidence of 0.5-1.5% and a lifetime incidence of 15-20% for CCA in PSC.[2–5] Increased risk of other malignancies, including gallbladder carcinoma (GBC), hepatocellular carcinoma (HCC) and colorectal carcinoma is also observed, but CCA predominates and is responsible for a large proportion of PSC-related deaths.[1–3,6]

In PSC, low-grade dysplasia (LGD) and high-grade dysplasia (HGD) in the bile ducts often precede CCA through a dysplasia–carcinoma sequence.[7] The rate and time interval for transformation from dysplasia to invasive CCA in PSC is largely unknown.[7–9] Malignant transformation in the bile duct is difficult to distinguish from benign disease progression in PSC as symptoms and imaging features poorly differentiate benign from malignant bile duct strictures.[1,10] Bile duct brush cytology and/or biopsies are indispensable to secure a diagnosis of dysplasia or CCA,[11] but are limited by low sensitivity and modest specificity in PSC.[12] Histologically, bile duct dysplasia has been reported in about one-third of liver explants of individuals with PSC.[7] In contrast to HCC, which is strongly linked to cirrhosis, bile duct dysplasia and CCA in PSC have not been clearly associated with advanced liver fibrosis.[7,13,14]

Evidence-based surveillance for CCA in PSC has not been established, but current guidelines recommend MRI/magnetic resonance cholangiopancreatography and/or ultrasound of the liver at regular intervals.[8,15] In cases of high-grade strictures on imaging, second-line evaluation with endoscopic retrograde cholangiography (ERC) and bile duct

* Corresponding author. Address: Section of Gastroenterology, Department of Transplantation Medicine, Division of Surgery and Specialized Medicine, Oslo University Hospital Rikshospitalet, Postboks 4950 Nydalen, N-0424 Oslo, Norway; Tel.: +4723070000.
E-mail address: trine.folseraas@medisin.uio.no (T. Folseraas).
† These authors contributed equally to this work and shared co-first authorship
https://doi.org/10.1016/j.jhepr.2025.101598

brushings from relevant strictures are often performed.[16,17] If bile duct dysplasia is diagnosed by brush cytology, repeated ERC with brush cytology to confirm the initial finding can improve diagnostic accuracy.[18] Single-operator cholangioscopy-guided targeted intraductal biopsies may further increase diagnostic accuracy.[18,19]

Liver transplantation (LT) is the only potential curative treatment for PSC,[1] and the disease is among the leading indications for LT in the Nordic countries.[20] Individuals with PSC are mainly considered for LT due to end-stage liver disease, symptoms from widespread or pronounced biliary strictures or suspected bile duct neoplasia.[8] Individuals with PSC and bile duct dysplasia are at some centers recognized as candidates for preemptive LT to avoid future, potentially incurable CCA.[18,21,22] This practice was introduced in Norway and Sweden in the early 2000s, and was rationalized by the poor prognosis of advanced CCA and the inability to diagnose early CCA, combined with short LT waitlist time and low waitlist mortality.[17,20,21,23,24]

The aim of this study was to evaluate our practice of preemptive LT in individuals with PSC and bile duct dysplasia to inform and potentially refine future criteria for selecting candidates for LT.

Materials and methods

Study design and study population

In this retrospective observational study individuals with PSC were identified based on their listing diagnoses of PSC or CCA in the Nordic Liver Transplant Registry (NLTR), and national PSC databases were used as a supplement to enhance case identification. Clinical, biochemical, radiological and histopathological data were collected from medical records and PSC databases in Norway and Sweden. Individuals who received their first LT between January 1st 2000 and December 31st 2021 at an age of ≥18 years were included (Fig. 1).

PSC and inflammatory bowel disease (IBD) were diagnosed according to standard criteria.[1,25] Clinical and biochemical status at the time of listing for LT, results of radiological examinations, bile duct brush cytology and biopsies performed prior to listing and results of histopathological evaluations of the explanted livers were recorded. Postoperative complications following LT were classified as early (<3 months from LT) or late (3-12 months from LT). Early complications were categorized into acute rejection, arterial thrombosis, arterial stenosis, bleeding, bile duct leakage, bile duct stenosis or other. While late complications were registered as any rejection, infection, bile duct stenosis, arterial stenosis or other. Recurrence of PSC was evaluated by retrospective review of the medical records, and based upon findings in liver biopsy, magnetic resonance cholangiopancreatography, ERC and/or histopathology at re-transplantation. Cause of death was registered using medical records. Mortality and re-transplantation data were collected until December 31st 2022.

Results of bile duct brush cytology were classified as: non-diagnostic (scarce material), non-neoplastic (normal epithelium, inflammatory or reactive changes), LGD or HGD. Ambiguous cytology diagnosis of irregular/atypical cells was classified as non-neoplastic, whereas suspicious LGD or HGD were classified as LGD or HGD, respectively. If multiple bile duct brush cytology samples had been obtained longitudinally at different time points, we registered the three most recent up to listing for LT within the last 3 years prior to listing. If multiple bile duct brush cytology samples were obtained from the same procedure, the highest reported grade of dysplasia was used in the statistical analysis. Fluorescence *in situ* hybridization (FISH) was available in only a small proportion of individuals, and hence not analysed. Bile duct dysplasia in the explant liver was registered according to the 2019 World Health Organization biliary intraepithelial neoplasia (BilIN) classification of tumours of the digestive system, where for the purpose of the statistical analysis BilIN-1 and BilIN-2 were recategorized as LGD and BilIN-3 as HGD.[26] CCA was staged according to The American Joint Committee on Cancer (AJCC) TNM-system 8th edition.[27,28]

The described histopathology of the bile ducts in the explanted livers were categorized as: non-neoplastic, LGD, HGD, CCA or other malignancy. Stage of fibrosis in the surrounding liver parenchyma in the explant livers was dichotomized into liver fibrosis (fibrosis stage 1-3) and cirrhosis (stage 4) based on the description in the pathology report. Test performance of bile duct brush cytology and biopsy was evaluated by positive predictive values (PPVs), by comparing LGD and HGD findings in bile duct specimens to explant liver histology.

Primary indication for liver transplantation

The primary indication for LT was determined and categorized retrospectively as follows: (I) Low-grade bile duct dysplasia (LGD group), diagnosed in bile duct brush cytology and/or bile duct biopsy. (II) High-grade bile duct dysplasia (HGD group), diagnosed in bile duct brush cytology and/or bile duct biopsy. (III) CCA strongly suspected, based upon suspected mass forming lesion or periductal infiltrating CCA on radiology and/or histological finding of CCA. (IV) Other hepatobiliary cancers strongly suspected (HCC and GBC). (V) End-stage liver disease (cirrhosis) and/or decompensation. (VI) Debilitating PSC symptoms (recurrent bacterial cholangitis and/or pruritus and/or fatigue). (VII) Other/combined indications, for those individuals where one isolated primary indication could not be clearly defined.

Ethical approvals

Study protocols were approved by the Regional Committee for Medical Research Ethics South-Eastern Norway (REK 28290) and the Regional Ethics Committee in Stockholm (2023-00459-01). All research was conducted in accordance with the Declarations of Helsinki.

Statistical analysis

Continuous data are presented as mean ± SD or median (IQR), and categorical data as numbers with percentages, as appropriate. Continuous variables were compared using independent Student's *t* test, and categorical data by both the chi-square test and Fisher's exact test. For small sample sizes, only Fisher's exact test was applied. Confidence intervals were obtained using the exact binomial interval. Survival was calculated using the Kaplan-Meier method. Two-sided p values <0.05 were considered significant. Statistical analyses

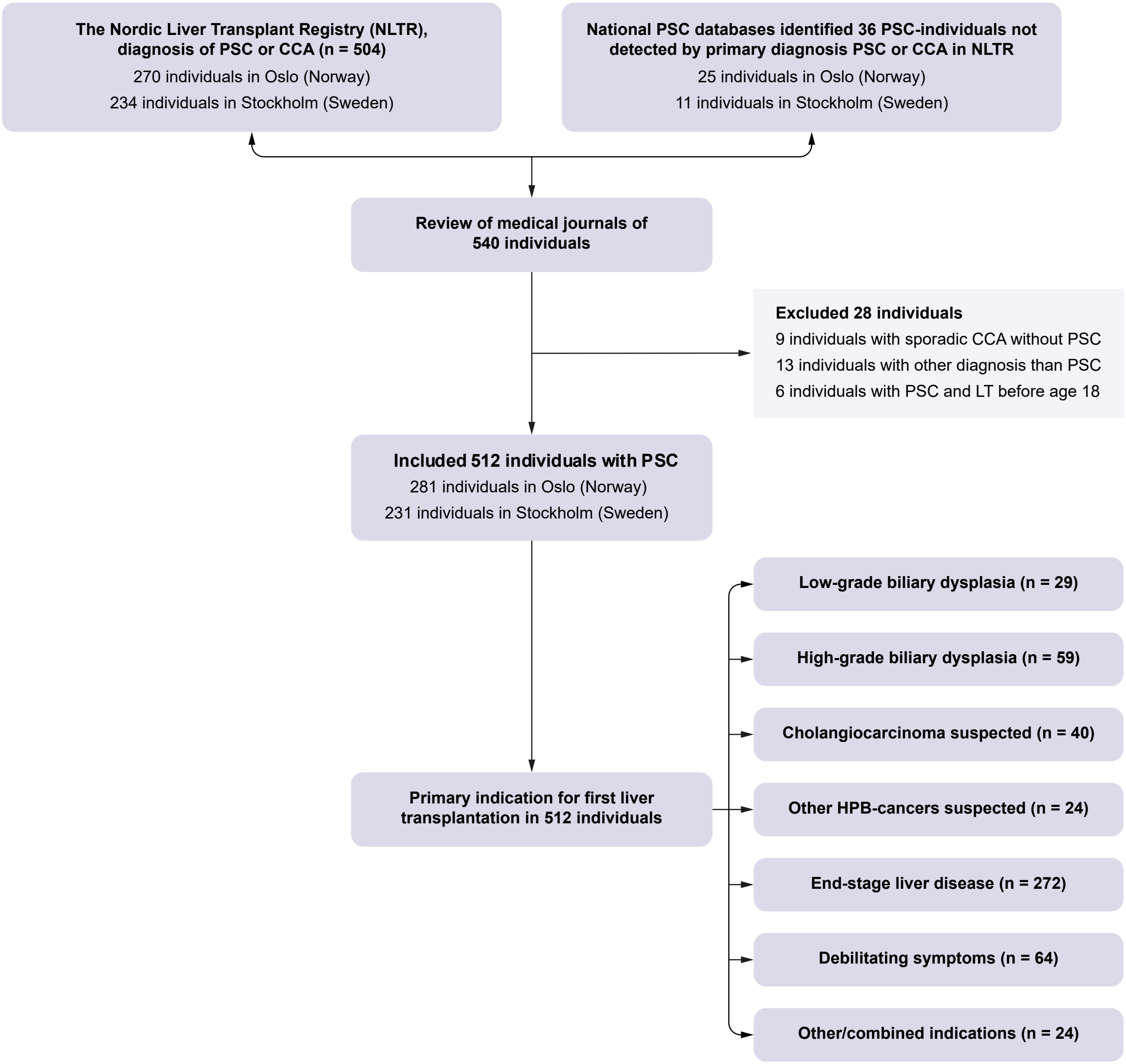

Fig. 1. Flowchart of individuals with diagnosis of PSC undergoing first LT from year 2000-2021. CCA, cholangiocarcinoma; LT, liver transplantation; NLTR, Nordic Liver Transplant Registry; PSC, primary sclerosing cholangitis.

were performed using STATA/SE version 17.0 (StataCorp LLC, TX, USA).

For the statistical analyses individuals listed for LT due to a primary indication of LGD or HGD were combined in one group, named "indication bile duct dysplasia" (n = 88). This group was compared to individuals with all other primary indications excluding suspicion of CCA, named "all other indications" (n = 384). Analyses were performed comparing age at first LT, IBD, MELD score, level of bilirubin and carbohydrate antigen 19-9, waiting time for LT, stage of liver fibrosis in explant liver histology and early and late postoperative complications following LT.

For evaluation of survival rate based on the primary indication for LT, we grouped the individuals as: (I) non-neoplastic indications, (II) LGD, (III) HGD or (IV) CCA. For comparisons, the non-neoplastic indications for LT constitute the combined grouping of all individuals with a primary indication of end-stage liver disease, debilitating symptoms and other/combined indications.

Results

Patient characteristics

In the 22-year study period, a total of 512 individuals with PSC received their first LT in Oslo (Norway, n = 281) and Stockholm (Sweden, n = 231). Most individuals were male (76%), had large-duct PSC (88%), and IBD (78%) at the time of listing for LT (Table 1). Mean age at the time of first LT was 45.5 ± 12.5 years, with a mean duration of PSC of 10.6 years before LT. The median MELD score across all indications was 13 (IQR 8-

Table 1. Demographic and clinical characteristics at time of listing for liver transplantation in individuals with primary sclerosing cholangitis (n = 512).

	All patients	Primary indication for listing to liver transplantation			
		Low-grade biliary dysplasia	High-grade biliary dysplasia	Suspicion of cholangiocarcinoma	All other indications
	(N = 512) (%)	(n = 29) (%)	(n = 59) (%)	(n = 40) (%)	(n = 384) (%)
Sex					
Male	391 (76.4)	23 (79.3)	43 (72.9)	31 (77.5)	294 (76.6)
Age at PSC diagnosis, years (mean)	34.9 ± 13.5	36.8 ± 11.6	35.4 ± 11.7	42.8 ± 13.2	33.9 ± 13.6
Age at IBD diagnosis, years (mean)	27.4 ± 13.5	29.5 ± 15.8	24.8 ± 10.8	30.0 ± 14.6	26.6 ± 13.1
Age at first LT, years (mean)	45.5 ± 12.5	43.4 ± 10.7	43.0 ± 12.0	49.8 ± 11.3	45.6 ± 12.7
Duration of PSC before LT, years (mean)	10.6	6.7	7.6	6.9	11.7
Subgroup of PSC					
Large-duct PSC	453 (88.5)	26 (89.7)	57 (96.6)	38 (95.0)	332 (86.4)
Small-duct PSC	4 (0.8)	0	0	1 (2.5)	3 (0.8)
PSC with features of AIH	55 (10.7)	3 (10.3)	2 (3.4)	1 (2.5)	49 (12.8)
IBD[†]	398 (77.9)	23 (79.3)	52 (88.1)	23 (57.5)	300 (78.3)
Prior colorectal neoplasia					
Low- or high-grade dysplasia	22 (4.3)	1 (3.4)	1 (1.7)	2 (5.0)	18 (4.7)
Cancer	14 (2.7)	2 (6.9)	2 (3.4)	0	10 (2.6)
No neoplasia	464 (90.6)	24 (82.8)	54 (91.5)	38 (95.0)	348 (90.6)
Unknown	12 (2.3)	2 (6.9)	2 (3.4)	0	8 (2.1)
Prior colectomy[††]	68 (13.3)	6 (21.4)	8 (13.6)	3 (7.5)	51 (13.3)
Prior cholecystectomy[†††]	81 (16.0)	5 (17.2)	10 (17.2)	6 (15.0)	60 (15.9)
Bilirubin (μmol/L) at time of listing (IQR)[‡]	52 (21-134)	11 (7-18)	16 (9-29)	31.5 (15.5-105)	71 (34-156)
CA 19-9 (kU/L) at time of listing (IQR)[‡‡]	32 (12-84)	14 (7-33)	16.5 (8-39)	120 (27.5-344)	34 (13-83)
MELD score at time of listing (IQR)[‡‡‡]	13 (8-18)	7 (6-8)	7 (6-9)	9 (7-15)	14 (11-19)
≤14	307 (60.4)	28 (96.6)	55 (93.2)	29 (72.5)	195 (51.3)
≥15	201 (39.6)	1 (3.4)	4 (6.8)	11 (27.5)	185 (48.7)

AIH, autoimmune hepatitis; CA 19-9, carbohydrate antigen 19-9; IBD, inflammatory bowel disease; LT, liver transplantation; MELD, model for end-stage liver disease; PSC, primary sclerosing cholangitis.

Values are presented as n (%), median (IQR) or mean ± SD. All percentages are presented as valid percent.

Indication bile duct dysplasia (LGD and HGD; n = 88) was compared with indication all other (n = 384). Using Student's *t* test significant differences were found in level of bilirubin (p <0.01) and MELD score (p <0.01), but no significant differences in age at first LT (p = 0.097) or level of CA 19-9 (p = 0.22). Using chi-square test, no difference in presence of IBD (p = 0.41).

[†]IBD: Missing information in one patient from all other indications.

[††]Prior colectomy: Missing information in two patients (indication LGD [n = 1], and all other indications [n = 1]).

[†††]Prior cholecystectomy: Missing information in seven patients (indication HGD [n = 1], and all other indications [n = 6]).

[‡]Bilirubin: Missing information in two patients from all other indications.

[‡‡]CA 19-9: Missing information in 39 patients (total) (LGD group [n = 2], HGD group [n = 3], suspected CCA group [n = 4], all other indications [n = 30]. The upper reference limit of CA 19-9 has experienced slight variations over time due to differences in analytical methods.

[‡‡‡]MELD: Missing information in four patients from all other indications.

18). For individuals transplanted due to LGD and HGD the median MELD score was 7 for both groups, respectively (IQR 6-8 in LGD and IQR 6-9 in HGD), while individuals transplanted due to end-stage liver disease had a median MELD score of 15 (IQR 12-20). Median CA 19-9 across all indications was 32 (IQR 12-84), while individuals transplanted due to LGD or HGD had median levels of CA 19-9 of 14 (IQR 7-33) and 16.5 kU/L (IQR 8-39), respectively (Table 1). Cirrhosis in the explanted liver was less frequent in individuals transplanted due to a primary indication of bile duct dysplasia 33/88 (37.5%) than all other indications combined 324/380 (85.3%, p <0.01) (Table 2). Mean waiting time for LT for the entire cohort was 87.6 days, increasing from a mean of 73 days (95% CI 61.5-84.0) in the first half (year 2000-2010) to a mean of 97 days (95% CI 85.1-108.0) in the second half (year 2011-2021) of the study period (p <0.01, data not shown). See Table 1 for a more detailed description of the cohort.

Primary indication for LT in PSC

Bile duct dysplasia was the primary indication for LT in 88/512 individuals (17.2%), of whom 29 (5.7%) had LGD and 59 (11.5%) HGD. Additionally, 40 (7.8%) were listed due to suspicion of CCA and 24 (4.7%) due to suspicion of GBC or HCC. Altogether, 152 individuals (29.7%) were listed with a primary indication of either bile duct dysplasia or strong suspicion of hepatobiliary malignancy. For the remaining individuals, the primary indication was end-stage liver disease and/or decompensation in 272 (53.1%), debilitating symptoms in 64 (12.5%) and other/combined indications in 24 (4.7%) (Fig. 1).

Characteristics of individuals with PSC listed for LT due to bile duct dysplasia

Individuals listed for LT due to bile duct dysplasia were slightly younger (mean age 43.4 ± 10.7 and 43.0 ± 12.0 years in the LGD group and HGD group, respectively) than individuals transplanted for all other indications combined (mean age 45.6 ± 12.7 years) (p = 0.097, LGD+HGD *vs*. all other). They also had significantly lower levels of bilirubin (p <0.01) and lower MELD scores (p <0.01) (Table 1). CA 19-9 levels and presence of IBD did not differ significantly compared to the other LT indications (p = 0.22 and p = 0.41 respectively).

Table 2. Histopathological findings in the explanted liver and bile ducts in individuals with primary sclerosing cholangitis (N = 512).

	All patients	Primary indication for listing to liver transplantation			
		Low-grade biliary dysplasia	High-grade biliary dysplasia	Suspicion of cholangiocarcinoma	All other indications
	(N = 512) (%)	(n = 29) (%)	(n = 59) (%)	(n = 40) (%)	(n = 384) (%)
Stage of liver fibrosis[†]					
Cirrhosis	367 (72.2)	10 (34.5)	23 (39.0)	10 (25.0)	324 (85.3)
Fibrosis stage 1-3	141 (27.8)	19 (65.5)	36 (61.0)	30 (75.0)	56 (14.7)
Bile duct neoplasia[††]					
No neoplasia	345 (68.3)	12 (41.4)	14 (23.7)	11 (27.5)[†††]	308 (81.7)
Low-grade dysplasia	86 (17.0)	14 (48.3)	17 (28.8)	2 (5.0)	53 (14.0)
High-grade dysplasia	29 (5.7)	1 (3.4)	19 (32.2)	0	9 (2.4)
Cholangiocarcinoma	45 (8.9)	2 (6.9)	9 (15.3)	27 (67.5)	7 (1.9)
Malignancy in hepatobiliary system[‡]					
No malignancy	438 (86.5)	27 (93.1)	50 (84.8)	12 (30.0)	349 (92.3)
Intrahepatic CCA	6 (1.2)	1 (3.45)	1 (1.7)	2 (5.0)	2 (0.5)
Perihilar CCA	35 (6.9)	0	8 (13.5)	22 (55.0)	5 (1.3)
Distal CCA	4 (0.8)	1 (3.45)	0	3 (7.5)	0
Hepatocellular carcinoma	17 (3.4)	0	0	0	17 (4.5)
Gallbladder carcinoma[‡‡]	5 (1.0)	0	0	1 (2.5)	4 (1.1)
Other malignancy[‡‡‡]	1 (0.2)	0	0	0	1 (0.3)

CCA, cholangiocarcinoma; LT, liver transplantation.
All percentages are reported as valid percentages (excluding missing values).
Significant difference in stage of liver fibrosis between groups.
Indication bile duct dysplasia (LGD and HGD; n = 88) was compared with indication all other (n = 384), chi-square test (p <0.01).
[†]Stage of liver fibrosis: Missing information in four patients from all other indications.
[††]Bile duct neoplasia: Missing information in seven patients from all other indications.
[†††]One patient with no neoplasia in the bile duct had findings of gallbladder carcinoma.
[‡]Malignancy in hepatobiliary system: Missing information in six patients from all other indications.
[‡‡]See Table S3 for further details on gallbladder neoplasia.
[‡‡‡]Other malignancy: One patient with finding of diffuse large B-cell lymphoma in liver.

Table 3. Overview of bile duct sampling prior to listing grouped by the primary indication for liver transplantation in individuals with primary sclerosing cholangitis (N = 512).

	All patients	Primary indication for listing to liver transplantation			
		Low-grade biliary dysplasia	High-grade biliary dysplasia	Suspicion of cholangiocarcinoma	All other indications
	(N = 512) (%)	(n = 29) (%)	(n = 59) (%)	(n = 40) (%)	(n = 384) (%)
**Brush cytology before listing with result LGD or HGD**	**146/512 (28.5)**	**28/29 (96.6)**	**57/59 (96.6)**	**22/40 (55.0)**	**39/384 (10.2)**
Finding of low-grade dysplasia in[†]					
One brush cytology sample	66 (12.9)	15/29 (51.7)	10/59 (17.0)	9/40 (22.5)	32/384 (8.3)
Two brush cytology samples	14 (2.7)	10/29 (34.5)	1 (1.7)	0	3/384 (0.8)
Three brush cytology samples	3 (0.6)	3 (10.3)	0	0	0
Finding of high-grade dysplasia in					
One brush cytology sample	47 (9.2)	0	30/59 (50.9)	10 (25.0)	7/384 (1.8)
Two brush cytology samples	31 (6.1)	0	23/59 (39.0)	5 (12.5)	3/384 (0.8)
Three brush cytology samples	3 (0.6)	0	3 (5.1)	0	0
**Bile duct biopsy before listing with result LGD, HGD or cancer**	**21/512 (4.1)**	**2/29 (6.9)**	**14/59 (23.7)**	**2/40 (5.0)**	**3/384 (0.8)**
Finding of low-grade dysplasia in					
One bile duct biopsy	10/512 (2.3)	1 (3.4)	5/59 (8.5)	1/40 (2.5)	3/384 (0.8)
Two bile duct biopsies	2/512 (0.4)	1 (3.4)	1/59 (1.7)	0	0
Finding of high-grade dysplasia in					
One bile duct biopsy	8/512 (1.6)	0	8/59 (13.6)[‡]	0	0
Finding of cancer in					
One bile duct biopsy	1/512 (0.2)	0	0	1/40 (2.5)	0

HGD, high-grade dysplasia; LGD, low-grade dysplasia.
Descriptive table: no statistical methods used to compare groups.
[†]In patients with a finding of LGD in brush cytology, 65/83 had only a finding of LGD, while 18/83 also had ≥1 brush cytology with HGD.
[‡]One patient in the HGD group had both one biopsy with finding of HGD and one biopsy with finding of LGD, and one patient had one bile duct biopsy with finding of HGD, without having any brush cytology samples with findings of dysplasia.

Details on cytological and histological findings in individuals listed for LT due to bile duct dysplasia are given in Table 3. In the LGD group 15/29 (51.7%) individuals had one brush cytology and 13/29 (44.8%) had two or more brush cytology results available prior to listing. In the HGD group, 30/59 (50.9%) had one brush cytology and 26/59 (44.1%) had two or more brush cytology results with HGD prior to listing. Repeated (≥2) bile duct brush sampling became more frequent throughout the study period, increasing from 30% to 55% in the LGD group and 40% to 49% in the HGD group in the period 2000-2010 to 2011-2021, respectively. Bile duct biopsies revealed dysplasia in 21 individuals (4.1%) in the entire cohort (Table 3). Of these, 16 individuals were listed for LT due to bile duct dysplasia and two were listed due to suspicion of CCA. Dysplasia was identified in bile duct biopsies in 2/29 (6.9%) of the individuals in the LGD group and in 14/59 (23.7%) in the HGD group (of which the biopsy had findings of HGD in 8/14 and LGD in 6/14). Of the eight individuals with HGD in bile duct biopsies, HGD or CCA was found in the explant liver in 6/8 (75%). All intraductal bile duct biopsies with findings of dysplasia in the HGD group were performed in the last half of the study period (year 2011-2021).

PPVs for detecting HGD or CCA in the explant liver varied by diagnostic method and number of specimens. For individuals with LGD as the primary LT indication, PPVs were 13.3% (95% CI 2%-40%) and 7.7% (95% CI 0.2%-36%) based on 1 or ≥2 brush cytology specimens, respectively. For those with HGD as the indication, PPVs increased to 33.3% (95% CI 17–53%) and 61.5% (95% CI 41–80%) based on 1 or ≥2 brush cytology specimens, respectively. Intraductal bile duct biopsy showing HGD (n = 8) yielded a PPV of 75% (95% CI 35–97%), while combining brush cytology and biopsy both showing HGD (n = 5) further increased PPV to 80% (95% CI 28–99%) for detecting HGD or CCA in the explant liver.

Histopathological findings in the explant livers

Bile duct neoplasia was described in the histopathological evaluation in 160/505 (31.7%) of explanted livers (Table 2). The correlation between primary indication for LT and the routine histopathological findings of the explanted liver bile ducts is presented in Fig. 2.

In individuals with bile duct dysplasia as the primary indication for LT, bile duct dysplasia or CCA could not be confirmed in the explant liver in 12 (41.4%) individuals with LGD and 14 (23.7%) individuals with HGD (p = 0.14).

Subgroup analyses of individuals listed due to LGD showed that those with HGD or CCA (n = 3) in the explanted liver were significantly older at time of PSC diagnosis (mean age 55.0 *vs.* 34.7, p <0.01) and at first LT (mean age 58.3 *vs.* 41.6, p <0.01) compared to individuals with no neoplasia or only LGD in the explant liver (n = 26). No other statistically significant differences were observed.

CCA was found in 45/505 (8.9%) of explant livers. In the subset of individuals with a primary LT indication of LGD or HGD, CCA was found in 2/29 (6.9%) and 9/59 (15.3%),

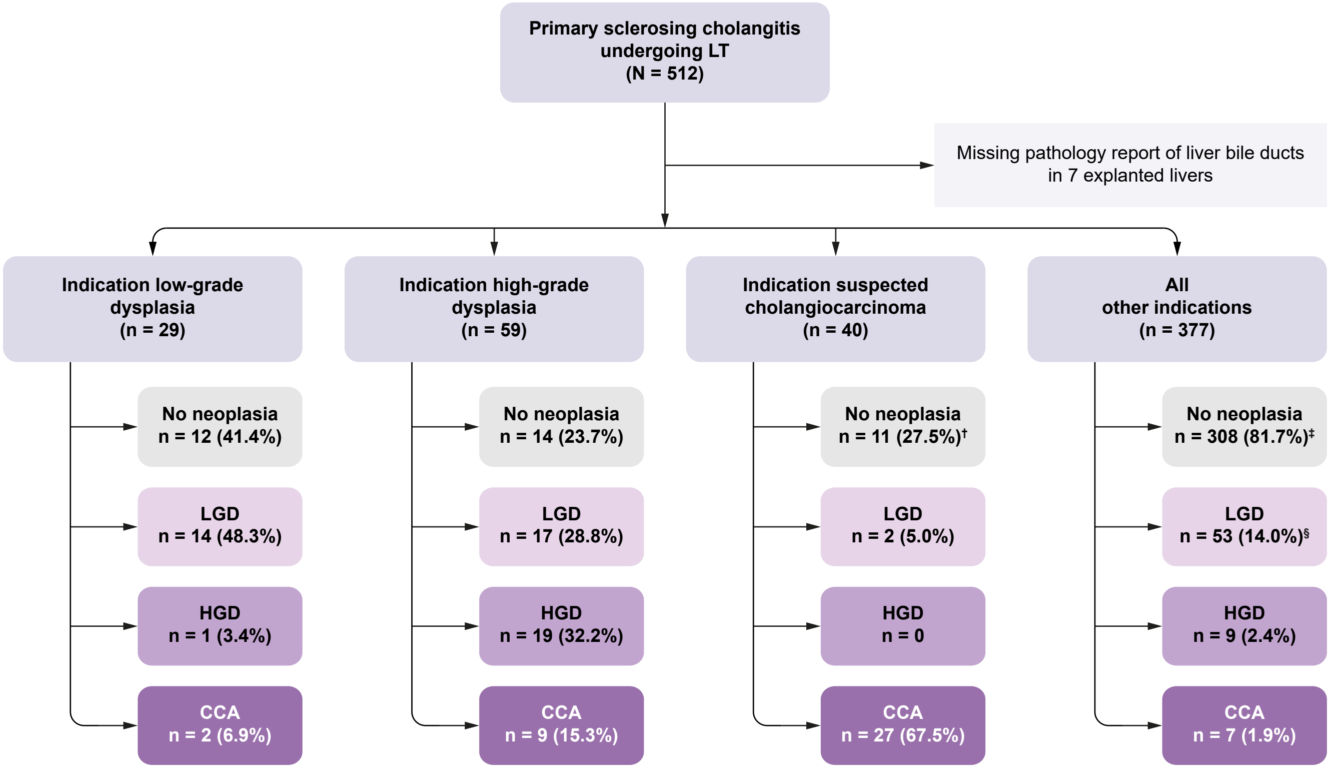

Fig. 2. The correlation between primary LT indications and histopathological findings in the bile ducts of explanted livers in individuals with primary sclerosing cholangitis (N = 512). †One patient in the indication suspected CCA group had findings of GBC (with no neoplasia in the bile ducts). ‡Two patients had findings of GBC. §One patient had findings of GBC. CCA, cholangiocarcinoma; GBC, gallbladder carcinoma; HGD, high-grade dysplasia; LGD, low-grade dysplasia; LT, liver transplantation.

respectively (p = 0.33). In individuals transplanted due to suspicion of CCA, CCA was found 27/40 (67.5%) and GBC in 1/40 (2.5%) of explants. CCA was found incidentally in five explant livers, of which four were found in the group of individuals with end-stage liver disease as the transplant indication (4/272, 1.5%) and one with debilitating symptoms as the transplant indication (1/64, 1.6%). HCC was found in 17/505 (3.4%) of the explant livers of which all had histological cirrhosis. Cirrhosis was present in 367/508 (72.2%) of all explant livers.

Waiting time, postoperative complications and re-transplantation

The mean time from listing to LT was 94.5 and 84.1 days in the LGD group and HGD group, respectively, compared with 88.8 days in all other indications combined (p = 0.91, LGD+HGD *vs.* all other, data not shown).

There was no difference in early and late postoperative complications following LT when comparing the indication bile duct dysplasia (LGD + HGD) to all other indications combined. During the study period, a re-transplantation was performed in 70 individuals (13.7%) a median of 5.0 years (range 0-17.3 years) following the first LT. The re-transplant rate in the bile duct dysplasia group was 9/88 (10.2%) and was not significantly different from all other indications (55/384, 14.3%) (p = 0.39).

Mortality and survival

At the end of the study period, 106/512 individuals (20.7%) had died. The most common cause of death was PSC-related malignancy (n = 34/106, 32.1%) and transplant complications (n = 24/106, 22.6%). The remaining deaths were because of other causes (n = 12/106, 11.3%), other malignancy (n = 9/106, 8.5%) and recurrent PSC (n = 3/106, 2.8%). The cause of death was unknown in n = 24/106 (22.6%). Causes of death, stratified by primary indication for LT, is presented in Table S1.

The survival distribution up to 10 years in PSC following LT according to the primary indication and the histopathological finding in the explant liver is shown in Fig. 3A,B. The overall 5-year and 10-year survival rates following LT in all individuals (N = 512) were 0.87 (95% CI 0.84-0.90) and 0.79 (95% CI 0.75-0.83). Based on the histopathological findings in the explanted liver, the 5-year and 10-year survival rate was 0.95 and 0.88 in no neoplasia, 0.89 and 0.86 in LGD, 0.85 and 0.73 in HGD, and 0.33 and 0.26 in CCA. Survival up to 10 years post-transplantation in individuals with PSC and CCA, stratified by AJCC-stage (0-4) is shown in Fig. S1.

Discussion

This study aimed to retrospectively evaluate the practice of preemptive LT in individuals with PSC and bile duct dysplasia, using real-world data from two large PSC cohorts in Norway and Sweden. The purpose was to inform and potentially refine future criteria for selecting candidates for LT in individuals with PSC-related bile duct dysplasia.

An important finding in our study was that in 30% of individuals listed for LT due to a primary indication of bile duct dysplasia in PSC, neither dysplasia nor CCA could be identified in the explanted liver. This proportion was even higher, at 41%, in the subgroup of individuals with LGD as the primary indication. Moreover, only 10% of individuals in the LGD group had findings of HGD or CCA in the explant liver, suggesting that accelerated progression to HGD or CCA is relatively uncommon in LGD. In contrast, in individuals with preoperative HGD, the absence of dysplasia in the explant was less frequent, but still notable, at 24%. Findings of HGD or CCA were confirmed in 48% of individuals listed due to HGD, indicating a higher, but not definitive correlation between preoperative diagnosis and histopathological confirmation in HGD. Bile duct dysplasia represented the second most frequent LT indication in PSC after end-stage liver disease during the study period, accounting for 17% of PSC-related transplants, thereby exerting notable impact on the transplant program capacity and waiting list.

The absence of histologically confirmed bile duct dysplasia in a substantial proportion of explants from individuals transplanted for suspected biliary dysplasia raises concerns regarding the practice of preemptive LT in dysplasia, particularly when LGD is the sole indication. Moreover, the frequent observation of "LGD only" in explant histology seen in 48% of individuals transplanted for LGD and 29% of those transplanted for HGD presents a challenge, especially given the potential reversibility of LGD and the uncertain rate and timeline of progression from LGD to HGD and CCA.[10] Our findings underscore the limitations of current diagnostic tools in accurately detecting premalignant lesions and progression of dysplasia in PSC. These limitations support the need for a carefully considered and selective approach to LT in individuals with LGD as the sole primary indication for LT. This is important to protect the individual from unnecessary surgical risk, lifelong immunosuppression and potential PSC recurrence, but also to ensure that donor organs are allocated to those individuals that will benefit the most. In contrast, HGD appears to represent a less questionable primary indication for LT. This is supported by previous studies identifying HGD as a stronger predictor than LGD for the presence of CCA[7,29] and by our data showing that HGD or CCA was confirmed in nearly 50% of explant livers from individuals listed for transplantation due to HGD. Ultimately, an individualized assessment of the potential benefits and risks of LT in cases where bile duct dysplasia is the sole indication should be conducted in consultation with the patient and following a multidisciplinary team discussion prior to making a final decision regarding listing.

Non-consistent findings of dysplasia in bile duct brush cytology prior to LT compared to the explant liver may have several explanations. Bile duct cytology evaluations are known to be difficult and inaccurate in both detecting and grading bile duct dysplasia in PSC.[12,30–32] Particularly, LGD may be difficult to distinguish from reactive, inflammatory changes in cytology while HGD may be difficult to differentiate from both lower grades of dysplasia and invasive CCA.[22,33] In addition, LGD is likely to be reversible and may therefore resolve by the time of LT.[34] In some cases, the histological examination of the explant liver may have been false negative for bile duct dysplasia, depending on the size and number of the tissue sections evaluated,[7] as well as the technical quality of bile ducts in the explant liver.

Individuals undergoing LT for bile duct dysplasia were generally younger, had a shorter duration of PSC and exhibited less advanced liver disease compared to individuals

A

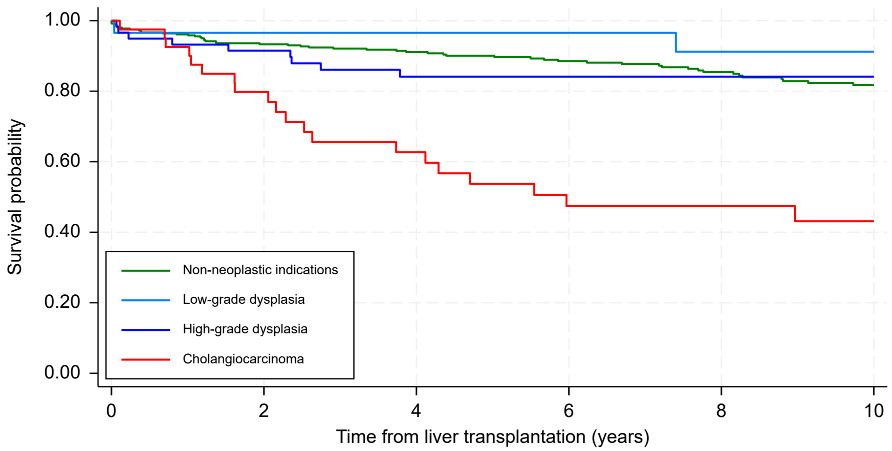

No. at risk						
Non-neoplastic	360	318	267	225	175	141
LGD	29	27	24	21	16	12
HGD	59	51	42	31	26	23
CCA	40	29	22	14	11	10

B

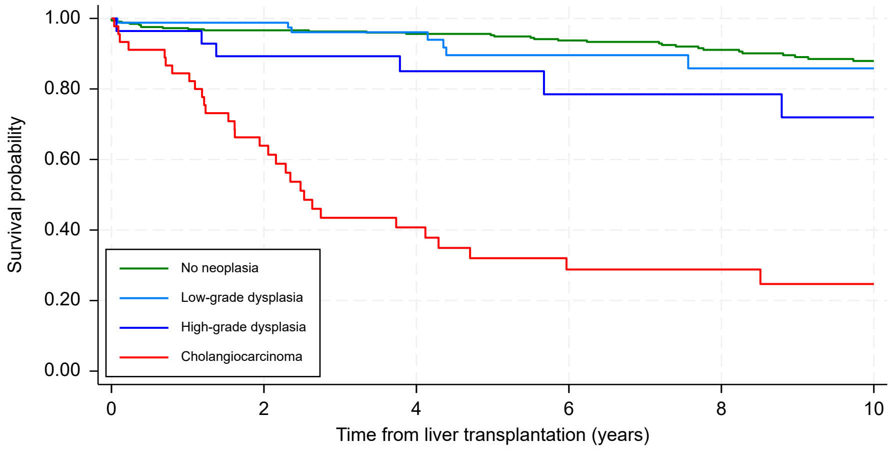

No. at risk						
No neoplasia	327	301	274	241	187	154
LGD	83	75	49	30	23	17
HGD	28	24	19	12	12	10
CCA	45	26	15	9	8	6

Fig. 3. Kaplan-Meier estimates of 10-year survival in primary sclerosing cholangitis following liver transplantation. (A) Survival based on primary indication for liver transplantation: Non-neoplastic indications: End-stage liver disease, debilitating symptoms, other/combined indications. Indication other hepatobiliary cancers are excluded (n = 24). (B) Survival based on histopathological findings in explanted liver bile ducts. Hepatocellular carcinoma, gallbladder carcinoma and other cancers are excluded (n = 29). HGD, high-grade dysplasia; LGD, low-grade dysplasia.

transplanted for end-stage disease. Despite these more favorable baseline characteristics, the frequencies of early and late postoperative complications, as well as recurrent PSC were comparable across the different transplant indications. This suggests that a lower pre-transplant risk profile of patients with dysplasia does not necessarily translate into reduced post-transplant morbidity. Long-term survival was favorable across the PSC cohort with a 10-year survival rate of 79%, in line with reports from the European Liver Transplant Registry.[35] In the subgroup transplanted for bile duct dysplasia, survival rates were even higher (91% for LGD and 84% for HGD, respectively), likely reflecting younger age and less advanced liver disease in these individuals at the time of LT. While these survival outcomes are encouraging, the decision to transplant for LGD must carefully balance the potential for excellent long-term survival against the risk of post-transplant complications and morbidity.

Enhanced diagnostic precision is essential to optimize the selection of candidates for resection or LT and to prevent progression to advanced, incurable CCA in PSC. To achieve this, more effective diagnostic strategies and accurate tools are needed to detect premalignant lesions and early-stage CCA in PSC. Current diagnostic strategies include use of bile duct brush cytology, FISH and single-operator cholangioscopy-guided intraductal biopsies. Repeated bile duct brushings over time have been associated with improved diagnostic accuracy for detecting dysplasia,[18] while single-operator cholangioscopy facilitates targeted biopsies and access to

strictures that may otherwise be inaccessible.[31] FISH is used as a complement to biliary brush cytology in many centers and has demonstrated potential to improve diagnostic accuracy in detecting CCA.[8,15,36,37] In our study, the increased use of repeated brush sampling in cases of LGD, aiming to more precisely diagnose dysplasia and monitor progression to HGD, appears meaningful given the presence of HGD and CCA in the explant livers of a subset of individuals listed due to LGD. This approach, along with increasing use of single-operator cholangioscopy-guided biopsies in the latter part of the study period, reflects an attempt to achieve more precise diagnostic evaluation prior to LT.

Despite ongoing improvements in diagnostic techniques, an urgent need persists for developing novel molecular biomarkers that enable early and accurate detection of premalignant and early-stage CCA in PSC.[38–40] Moreover, the molecular and histopathological events that drive the progression from dysplasia to invasive carcinoma remain poorly defined. A clearer understanding of these events could guide the development of reliable biomarkers for early malignant transformation and optimize the timing of LT in PSC.

The strengths of our study include a large cohort of individuals with PSC, comprehensive clinicopathological data and long-term follow-up. However, several limitations should be acknowledged. The retrospective design of the study in some cases introduced uncertainty in determining the primary indication for LT, as decisions may have been influenced by several factors and individual assessments. Additionally, histopathological evaluations were based on routine reports, in which bile duct dysplasia may not have been systematically examined for, potentially leading to underreporting. Differences over time, including changes in the pre-transplant evaluation, histopathological interpretation and imaging quality, and differences between the two LT centers are not fully accounted for in this study.[41]

In conclusion, our findings support a thoughtful and individualized approach when considering LT in patients with LGD, to avoid premature or unnecessary transplantation and to minimize the impact on waiting list resources. In contrast, HGD, when accurately diagnosed, appears to be a clearly justifiable indication for LT, given its strong association with early CCA in the explant and the extremely poor prognosis of advanced disease. To optimize candidate selection for LT in PSC, continued efforts to clarify the dysplasia–carcinoma sequence and to develop more accurate diagnostic tools for premalignant and early-stage CCA are warranted.

Affiliations

[1]*Institute of Clinical Medicine, Faculty of Medicine, University of Oslo, Oslo, Norway;* [2]*Norwegian PSC Research Center, Department of Transplantation Medicine, Division of Surgery and Specialized Medicine, Oslo University Hospital Rikshospitalet, Oslo, Norway;* [3]*Department of Transplantation Surgery, Karolinska University Hospital, Stockholm, Sweden;* [4]*Department of Medicine Huddinge, Karolinska Institutet, Stockholm, Sweden;* [5]*Section of Gastroenterology, Department of Transplantation Medicine, Division of Surgery and Specialized Medicine, Oslo University Hospital, Rikshospitalet, Oslo, Norway;* [6]*Department of Pathology, Oslo University Hospital, Rikshospitalet, Oslo, Norway;* [7]*Research Institute of Internal Medicine, Division of Surgery and Specialized Medicine, Oslo University Hospital, Rikshospitalet, Oslo, Norway;* [8]*Hybrid Technology Hub Centre of Excellence, Institute of Basic Medical Sciences, Faculty of Medicine, University of Oslo, Oslo, Norway;* [9]*Section of Transplant Surgery, Department of Transplantation Medicine, Division of Surgery and Specialized Medicine, Oslo University Hospital Rikshospitalet, Oslo, Norway;* [10]*Department of Upper Abdominal Diseases, Karolinska University Hospital, Stockholm, Sweden*

Abbreviations

BilIN, biliary intraepithelial neoplasia; CCA, cholangiocarcinoma; ERC, endoscopic retrograde cholangiography; FISH, fluorescence *in situ* hybridization; GBC, gallbladder carcinoma; HCC, hepatocellular carcinoma; HGD, high-grade dysplasia; IBD, inflammatory bowel disease; LGD, low-grade dysplasia; LT, liver transplantation; MELD, model for end-stage liver disease; NLTR, the Nordic Liver Transplant Registry; PSC, primary sclerosing cholangitis.

Financial support

Sigurd Breder: Not applicable; Christina Villard: Not applicable; Emma Eide: Not applicable; Benny Wang: Not applicable; Lise Katrine Engesæter: South-Eastern Health authorities; Henrik Mikael Reims: Not applicable; Johannes Roksund Hov: Has received research support from Biogen and Pfizer and lecture honoraria from Amgen, Roche and Novartis; Espen Melum: Research council of Norway, South-Eastern Health authorities; Lars Aabakken: Not applicable; Pål Dag Line: Not applicable; Jon Lømo: Received advisory board fees from Daichi-Sankyo, Exact Sciences and Eli Lilly, and lecture fees from several companies (Gilead, Astra Zeneca, among others); Krzyztof Grzyb: Not applicable; Kristine Wiencke: Not applicable; Annika Bergquist: Swedish Cancer Society, Stockholm County Council; Trine Folseraas: Not applicable.

Conflict of interest

No conflict of interests in relation to this paper.

Please refer to the accompanying ICMJE disclosure forms for further details.

Authors' contributions

Study concept and design: AB and TF. Acquisition of histological and clinical data: SB, CV, EE, BW, LKE, HMR, JRH, EM, LA, PDL, JL, KG, KW, AB and TF. Analysis and interpretation of data: SB, CV, AB and TF. Statistical analyses: SB and CV. Drafting of manuscript: SB, CV, AB and TF. All authors contributed to critical revision and final approval of the manuscript.

Data availability

The data supporting this study's findings are available from the corresponding author upon request.

Acknowledgments

We would like to express our gratitude to the European Reference Network on Rare Liver Diseases (ERN-Rare-Liver: https://rare-liver.eu/). We would also like to express gratitude to Sissel Akra, Biobank Manager at the Norwegian PSC Biobank. We are grateful for the efforts put down by transplant coordinators and clinicians at the involved centers ensuring that NLTR is updated. JRH was in part funded by ERC (no. 802544). LKE was funded by the Regional Health Authorities South-Eastern Norway (no. 2017022).

Supplementary data

Supplementary data to this article can be found online at https://doi.org/10.1016/j.jhepr.2025.101598.

References

Author names in bold designate shared co-first authorship

[1] Karlsen TH, Folseraas T, Thorburn D, et al. Primary sclerosing cholangitis - a comprehensive review. J Hepatol 2017;67(6):1298–1323.

[2] Bergquist A, Ekbom A, Olsson R, et al. Hepatic and extrahepatic malignancies in primary sclerosing cholangitis. J Hepatol 2002;36(3):321–327.

[3] Boonstra K, Weersma RK, van Erpecum KJ, et al. Population-based epidemiology, malignancy risk, and outcome of primary sclerosing cholangitis. Hepatology 2013;58(6):2045–2055.

[4] Dyson JK, Beuers U, Jones DEJ, et al. Primary sclerosing cholangitis. Lancet 2018;391(10139):2547–2559.

[5] Karlsen TH, Schrumpf E, Boberg KM. Update on primary sclerosing cholangitis. Dig Liver Dis 2010;42(6):390–400.

[6] de Valle MB, Björnsson E, Lindkvist B. Mortality and cancer risk related to primary sclerosing cholangitis in a Swedish population-based cohort. Liver Int 2012;32(3):441–448.
[7] Lewis JT, Talwalkar JA, Rosen CB, et al. Precancerous bile duct pathology in end-stage primary sclerosing cholangitis, with and without cholangiocarcinoma. Am J Surg Pathol 2010;34(1):27–34.
[8] European Association for the Study of the Liver. EASL clinical practice guidelines on sclerosing cholangitis. J Hepatol 2022;77(3):761–806.
[9] Fevery J, Verslype C. An update on cholangiocarcinoma associated with primary sclerosing cholangitis. Curr Opin Gastroenterol 2010;26(3):236–245.
[10] Ilyas SI, Eaton JE, Gores GJ. Primary sclerosing cholangitis as a premalignant biliary tract disease: surveillance and management. Clin Gastroenterol Hepatol 2015;13(12):2152–2165.
[11] Aabakken L, Karlsen TH, Albert J, et al. Role of endoscopy in primary sclerosing cholangitis: European society of gastrointestinal endoscopy (ESGE) and European association for the study of the liver (EASL) clinical guideline. Endoscopy 2017;49(6):588–608.
[12] Navaneethan U, Njei B, Lourdusamy V, et al. Comparative effectiveness of biliary brush cytology and intraductal biopsy for detection of malignant biliary strictures: a systematic review and meta-analysis. Gastrointest Endosc 2015;81(1):168–176.
[13] **Zenouzi R**, **Weismüller TJ**, Hübener P, et al. Low risk of hepatocellular carcinoma in patients with primary sclerosing cholangitis with cirrhosis. Clin Gastroenterol Hepatol 2014;12(10):1733–1738.
[14] **Ali AH**, **Tabibian JH**, Nasser-Ghodsi N, et al. Surveillance for hepatobiliary cancers in patients with primary sclerosing cholangitis. Hepatology 2018;67(6):2338–2351.
[15] Bowlus CL, Arrivé L, Bergquist A, et al. AASLD practice guidance on primary sclerosing cholangitis and cholangiocarcinoma. Hepatology 2023;77(2):659–702.
[16] **Boyd S**, **Tenca A**, Jokelainen K, et al. Screening primary sclerosing cholangitis and biliary dysplasia with endoscopic retrograde cholangiography and brush cytology: risk factors for biliary neoplasia. Endoscopy 2016;48(5):432–439.
[17] Boberg KM, Jebsen P, Clausen OP, et al. Diagnostic benefit of biliary brush cytology in cholangiocarcinoma in primary sclerosing cholangitis. J Hepatol 2006;45(4):568–574.
[18] Majeed A, Castedal M, Arnelo U, et al. Optimizing the detection of biliary dysplasia in primary sclerosing cholangitis before liver transplantation. Scand J Gastroenterol 2018;53(1):56–63.
[19] Waldthaler A, Schramm C, Bergquist A. Present and future role of endoscopic retrograde cholangiography in primary sclerosing cholangitis. Eur J Med Genet 2021;64(6):104231.
[20] Engesæter LK, Melum E. The nordic liver transplant Registry (NLTR) annual report 2023. 2024 June 2024.
[21] **Andersen IM**, **Fosby B**, Boberg KM, et al. Indications and outcomes in liver transplantation in patients with primary sclerosing cholangitis in Norway. Transpl Direct 2015;1(9):e39.
[22] Boyd S, Vannas M, Jokelainen K, et al. Suspicious brush cytology is an indication for liver transplantation evaluation in primary sclerosing cholangitis. World J Gastroenterol 2017;23(33):6147–6154.
[23] Brandsaeter B, Broomé U, Isoniemi H, et al. Liver transplantation for primary sclerosing cholangitis in the Nordic countries: outcome after acceptance to the waiting list. Liver Transpl 2003;9(9):961–969.
[24] Boberg KM, Jebsen P, Clausen OP, et al. Cholangiocarcinoma in situ in primary sclerosing cholangitis: diagnosis by brush cytology and treatment by liver transplantation. J Hepatol 2003;39(3):453.
[25] Maaser C, Sturm A, Vavricka SR, et al. ECCO-ESGAR Guideline for Diagnostic Assessment in IBD Part 1: initial diagnosis, monitoring of known IBD, detection of complications. J Crohns Colitis 2019;13(2):144–164.
[26] Nagtegaal ID, Odze RD, Klimstra D, et al. The 2019 WHO classification of tumours of the digestive system. Histopathology 2020;76(2):182–188.
[27] Amin MB, Greene FL, Edge SB, et al. The Eighth Edition AJCC Cancer Staging Manual: continuing to build a bridge from a population-based to a more "personalized" approach to cancer staging. CA Cancer J Clin 2017;67(2):93–99.
[28] Chun YS, Pawlik TM, Vauthey JN. 8th edition of the AJCC cancer staging manual: pancreas and hepatobiliary cancers. Ann Surg Oncol 2018;25(4):845–847.
[29] Grimsrud MM, Folseraas T. Pathogenesis, diagnosis and treatment of premalignant and malignant stages of cholangiocarcinoma in primary sclerosing cholangitis. Liver Int 2019;39(12):2230–2237.
[30] Trikudanathan G, Navaneethan U, Njei B, et al. Diagnostic yield of bile duct brushings for cholangiocarcinoma in primary sclerosing cholangitis: a systematic review and meta-analysis. Gastrointest Endosc 2014;79(5):783–789.
[31] Arnelo U, von Seth E, Bergquist A. Prospective evaluation of the clinical utility of single-operator peroral cholangioscopy in patients with primary sclerosing cholangitis. Endoscopy 2015;47(8):696–702.
[32] Kaura K, Sawas T, Bazerbachi F, et al. Cholangioscopy biopsies improve detection of cholangiocarcinoma when combined with cytology and FISH, but not in patients with PSC. Dig Dis Sci 2020;65(5):1471–1478.
[33] Kamp E, Dinjens WNM, van Velthuysen MF, et al. Next-generation sequencing mutation analysis on biliary brush cytology for differentiation of benign and malignant strictures in primary sclerosing cholangitis. Gastrointest Endosc 2023;97(3):456. 65.e6.
[34] Tsen A, Atluri S, Henkes N, et al. Regression of low grade biliary dysplasia following endoscopic stent placement: a case report. 2017 October 2017. https://journals.lww.com/ajg/fulltext/2017/10001/regression_of_low_grade_biliary_dysplasia.2067.aspx/University/of/Texas/Health/Science/Center/at/San/Antonio/SA/Pathology/Reference/Laboratory/of/San/Antonio/SA.
[35] Heinemann M, Adam R, Berenguer M, et al. Longterm survival after liver transplantation for autoimmune hepatitis: results from the European liver transplant Registry. Liver Transpl 2020;26(7):866–877.
[36] von Seth E, Ouchterlony H, Dobra K, et al. Diagnostic performance of a stepwise cytological algorithm for biliary malignancy in primary sclerosing cholangitis. Liver Int 2019;39(2):382–388.
[37] Navaneethan U, Njei B, Venkatesh PG, et al. Fluorescence in situ hybridization for diagnosis of cholangiocarcinoma in primary sclerosing cholangitis: a systematic review and meta-analysis. Gastrointest Endosc 2014;79(6):943. 50.e3.
[38] Vedeld HM, Folseraas T, Lind GE. Detecting cholangiocarcinoma in patients with primary sclerosing cholangitis - the promise of DNA methylation and molecular biomarkers. JHEP Rep 2020;2(5):100143.
[39] Eaton JE, Vesterhus M, McCauley BM, et al. Primary sclerosing cholangitis risk estimate tool (PREsTo) predicts outcomes of the disease: a derivation and validation study using machine learning. Hepatology 2020;71(1):214–224.
[40] Macias RIR, Cardinale V, Kendall TJ, et al. Clinical relevance of biomarkers in cholangiocarcinoma: critical revision and future directions. Gut 2022;71(8):1669–1683.
[41] Bergquist A, Weismüller TJ, Levy C, et al. Impact on follow-up strategies in patients with primary sclerosing cholangitis. Liver Int 2023;43(1):127–138.

Keywords: Primary sclerosing cholangitis (PSC); Bile duct dysplasia; Cholangiocarcinoma (CCA); Liver transplantation (LT).

Received 19 June 2025; received in revised form 8 September 2025; accepted 16 September 2025; Available online 20 September 2025

Journal of Hepatology, Volume *7*

Supplemental information

A retrospective evaluation of preemptive liver transplantation for bile duct dysplasia in primary sclerosing cholangitis: Balancing risks and benefits

Sigurd Breder, Christina Villard, Emma Eide, Benny Wang, Lise Katrine Engesæter, Henrik Mikael Reims, Johannes Roksund Hov, Espen Melum, Lars Aabakken, Pål Dag Line, Jon Lømo, Krzyztof Grzyb, Kristine Wiencke, Annika Bergquist, and Trine Folseraas

A retrospective evaluation of preemptive liver transplantation for bile duct dysplasia in primary sclerosing cholangitis: Balancing risks and benefits

Sigurd Breder, Christina Villard, Emma Eide, Benny Wang, Lise Katrine Engesæter, Henrik Mikael Reims, Johannes Roksund Hov, Espen Melum, Lars Aabakken, Pål Dag Line, Jon Lømo, Krzyztof Grzyb, Kristine Wiencke, Annika Bergquist, Trine Folseraas

Table of contents

Fig. S1 2

Table S1 3

Fig. S1. The survival distribution in cholangiocarcinoma up to 10-years following liver transplantation divided according to stage

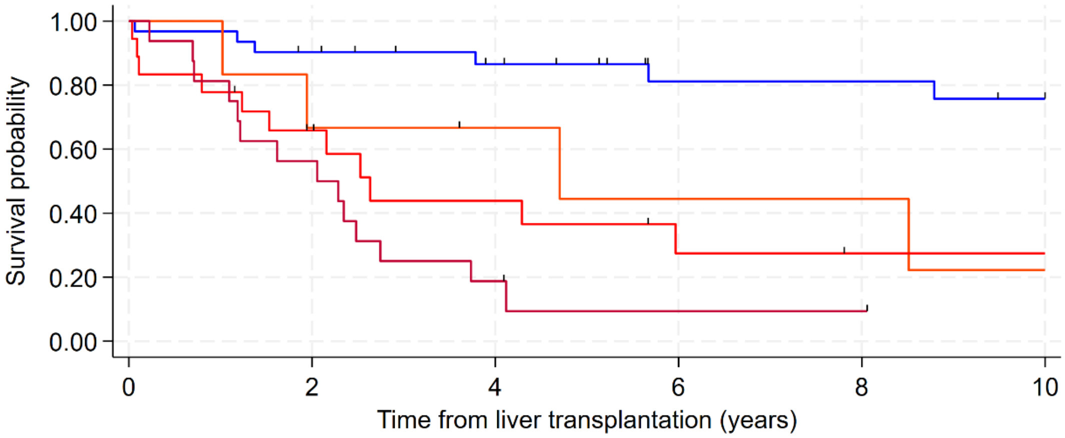

Number at risk

	0	2	4	6	8	10
HGD/Stage 0 CIS	31	27	22	15	15	13
CCA stage 1	6	4	3	2	2	1
CCA stage 2	18	9	6	3	2	2
CCA stage 3-4	16	9	3	1	1	0

CCA: Cholangiocarcinoma. HGD: High-grade dysplasia. CIS: Carcinioma in situ. Stage according to The American Joint Committee on Cancer (AJCC) TNM-system 8th edition

Table S1. Causes of death, stratified by primary indication for liver transplantation in primary sclerosing cholangitis

	All patients	Primary indication for listing to liver transplantation			
		Low-grade biliary dysplasia	High-grade biliary dysplasia	Suspicion of cholangiocarcinoma	All other indications
	(n = 512) (%)	(n = 29) (%)	(n = 59) (%)	(n = 40) (%)	(n = 384) (%)
**Dead (by December 31st 2022)**	106 (20.7)	3 (10.3)	12 (20.3)	21 (52.5)	70 (18.2)
**Age at death, years (range)**	54.8 (25.4-82.8)	48.8 (40.1-65.4)	54.5 (44.1-71.5)	54.8 (42.1-76.6)	54.8 (25.4-82.8)
**Time from LT to death, years (range)**	3.8 (0-22.5)	7.4 (0.04-18.6)	2.4 (0.07-19.3)	2.3 (0.1-13.9)	5.0 (0-22.5)
**Cause of death**					
PSC-related malignancy	34 (32.1)	0	5 (41.6)	14 (66.6)	15 (21.4)
Transplant complications	24 (22.6)	1 (33.3)	2 (16.7)	2 (9.5)	19 (27.1)
Other	12 (11.3)	1 (33.3)	2 (16.7)	3 (14.3)	6 (8.6)
Other malignancy	9 (8.5)	0	1 (8.3)	1 (4.8)	7 (10.0)
Recurrent PSC	3 (2.8)	0	0	0	3 (4.3)
Unknown	24 (22.7)	1 (33.3)	2 (16.7)	1 (4.8)	20 (28.6)

Values are presented as n (%), median (range). All percentages are presented as valid percent. LT: Liver transplantation.
